# How Pause Duration Influences Impressions of English Speech: Comparison Between Native and Non-native Speakers

**DOI:** 10.3389/fpsyg.2022.778018

**Published:** 2022-02-11

**Authors:** Shimeng Liu, Yoshitaka Nakajima, Lihan Chen, Sophia Arndt, Maki Kakizoe, Mark A. Elliott, Gerard B. Remijn

**Affiliations:** ^1^Department of Human Science, Faculty of Design, Kyushu University, Fukuoka, Japan; ^2^Sound Corporation, Fukuoka, Japan; ^3^School of Psychological and Cognitive Sciences, Peking University, Beijing, China; ^4^School of Psychology, National University of Ireland Galway, Galway, Ireland; ^5^Department Pädagogik und Rehabilitation, Fakultät für Psychologie und Pädagogik, Ludwig Maximilians Universität, Munich, Germany; ^6^Department of Acoustic Design, Faculty of Design, Kyushu University, Fukuoka, Japan

**Keywords:** speech, punctuation, pause duration, perceptual impression, factor analysis, rating scale

## Abstract

The purpose of this study was to investigate how the subjective impression of English speech would change when pause duration at punctuation marks was varied. Two listening experiments were performed in which written English speech segments were rated on a variety of evaluation items by both native-English speakers and non-native speakers (native-Chinese speakers and native-Japanese speakers). The ratings were then subjected to factor analysis. In the first experiment, the pauses in three segments were made into the same durations, from 0.075 to 4.8 s. Participants rated the segments on 23 evaluation items on a rating scale from 1 to 10. A varimax rotation after PCA (principal component analysis) led to two factors that were related to speech style. These two factors could be interpreted as representing speech naturalness and speech rate. Speech segments with a pause duration of 0.6 s received the highest naturalness evaluation, while perceived speech rate decreased as the physical pause duration increased, without any changes in utterance segments. In the second experiment, a full-factorial design of pause durations (0.15, 0.3, 0.6, 1.2, and 2.4 s) within and between sentences, i.e., for commas and for periods, was implemented in two speech segments. The original speech segments and speech segments without any pauses were also included as control conditions. From ratings on 12 evaluation items, similar to Experiment 1, two factors representing speech naturalness and speech rate were obtained. The results showed again that the perceived speech rate decreased with an increase only in pause duration. As for speech naturalness, the highest evaluations occurred when pause durations were 0.6 s within sentences, and either 0.6 or 1.2 s between sentences. This recommends fixing all pause durations to 0.6 s as a practical way to train non-native speakers to make their spoken English appear more natural.

## Introduction

Research into speech pause production and perception has a long tradition, with Tosi (1965) the first to introduce the word *pausology* in a study on speech and music. This was then adopted by O’Connell and Kowal (1983) who investigated silent pauses when reading aloud. O’Connell and Kowal identified various factors that could influence the voluntary use of pauses in speech, such as the speaker’s need to breathe, his/her emotional condition, the syntactic complexity of the text, the availability of lexical items, emphasis, and many others (see also Todd, 1985; Lucas, 2015; Barry, 2017). Pauses also play a role as turn-taking in communicative interactions (Sacks et al., 1974; Taneichi, 2014).

Pause duration varies with communication style (O’Connell and Kowal, 1983). For example, for story telling in English, a mean pause duration of 0.94 s (SD = 0.23 s) was found for segments with a minimum cut-off in between 0.20 and 0.31 s, including commas and periods. However, in interviews the mean pause duration was 0.53 s (SD = 0.06) (Kowal et al., 1983). For English and Spanish narratives, the mean pause durations were 0.69 and 0.73 s, respectively (de Johnson et al., 1979), while in poetry readings in English and German, the longest pause duration was used for punctuated line-ends, with a mean duration of 0.71 s [O’Connell and Kowal (1984)].

Mandatory pausing points are made at punctuation marks, which are used to give meaning and clarity to a sentence, or to separate phrases (Straus et al., 2014). Essentially, their main function is to group speech elements into units (Goldman-Eisler, 1972; Grosjean et al., 1979; Oliveira, 2002). Data from separate analyses of comma- and period-pause durations showed that pause durations between sentences (i.e., at periods) are longer than in between clauses within sentences (e.g., at commas; Cruttenden, 1986). For example, in oral deliveries of sermons in German, the mean duration for commas was 0.47 s (SD = 0.22), while for periods it was 0.98 s (SD = 0.34) [O’Connell and Kowal, 1986]. Interestingly, the average comma and period durations in four university commencement speeches in English were similar to these durations, i.e., 0.49 s (SD = 0.26) for commas and 1.01 s (SD = 0.40) for periods (Yamashita and Fuyuno, 2015). Finally, public presentations in English showed an average pause duration of 0.38 s (SD = 0.22) within sentences and of 0.98 s (SD = 0.33) between sentences. For another script, pause duration within and between sentences was 0.45 s (SD = 0.31) and 0.81 s (SD = 0.31), respectively (Yamashita et al., 2019). Taken together, the research on a variety of studies on pausing in speech has shown that the mean physical durations of commas (range: 0.38 to 0.67 s) and periods (range: 0.81 to 1.24 s) thus typically have a ratio of 1: 2.

Since English is used as a lingua franca (e.g., Jenkins et al., 2011), non-native speakers far outnumber native speakers (Crystal, 2008). For most beginning speakers of a language that is syntactically very different from their first language, it is a primary issue to learn how and when to pause, and to control pause durations. As Handel (1989) argued in his classic chapter on rhythm perception, to control pause durations is very important in speech communication. In preliminary studies on this topic with learners of English as a second language (L2 learners), recordings were obtained from students in EFL writing and speaking courses at two Japanese universities, who practiced English public presentations (Yamashita et al., 2014), or participated in a speech competition (Liu et al., 2016). Temporal factors in their speech were analyzed, including the number of pauses, their median duration and maximum duration, the standard deviation of the pause duration, and the coefficient of variations in pause duration within sentences (e.g., commas) and between sentences (e.g., periods). In these studies (Yamashita et al., 2014; Liu et al., 2016), the median pause duration ranged from 0.40 to 0.64 s. The maximum pause duration, however, varied considerably between speakers (1.15 to 4.49 s). The coefficient of variations of pause duration reflected the speaker’s proficiency: Participants who had a lower coefficient tended to get a higher evaluation in the speech competition (Liu et al., 2016). In the top-3 speeches with the highest evaluations, among a total of 11 speeches, the pause duration within sentences was 0.59, 0.42, and 0.67 s, while the pause duration between sentences was 1.24, 0.83, and 1.13 s, respectively. The pause durations between sentences and within sentences thus also had a ratio of about 1:2 for these proficient L2-leaners. By contrast, for the 3 bottom-ranked L2-learners the ratio varied considerably. Their pause duration within sentences ranged from 0.50 to 0.92 s, while it ranged between sentences from 0.86 to 1.54 s, thus with a ratio in between 1:1.72 to 1:2.5. In a related study, pause insertion patterns of English L2-learners were also investigated from a perspective of multimodal corpora (Fuyuno et al., 2016). The relative cumulative frequencies of the duration of pauses in commas and periods of proficient L2-learners were similar to those of native-English speakers. Furthermore, proficient L2-learners demonstrated a similar pause insertion pattern (Fuyuno et al., 2017). Proficient L2-learners also shared similar pause patterns (i.e., number, duration, and location of pauses) in different speech rates in speech production (Matzinger et al., 2020), and no difference in pause duration and distribution compared to their own languages (Black et al., 1966). Pause duration control thus should have contributed to the quality of L2-learners’ speaking performance.

The research on L2-learners’ use of pauses and that on the voluntary use of pauses by native speakers during public speaking (Lucas, 2015; Barry, 2017) strongly suggests that pause duration affects our general impression of speech. “Voluntary” here means that the speaker uses different pauses at different places to make his/her speech delivery impressive to the audience in public speaking. For example, the speaker can leave a relatively long pause at the end of a thought unit, to allow the audience to think. This way, pauses are used as a rehearsal time for short-term memory, especially for the listeners (Sugito, 1990). Barry (2017) also pointed out that the speaker’s job is to let the audience think rather than talk, and the only time for thinking is during pauses. So far, research on the perception of pauses in speech – rather than their production – has mainly focused on the perceptual under- or overestimation of pause durations (Stuckenberg and O’Connell, 1988), or on the automatic detection of pauses in speech with computers (Horii, 1983; Goto et al., 1999; Rosen et al., 2010). Little is known, however, on (1) how systematic changes in pause duration influence subjective impressions of English speech, and (2) whether favorable impressions occur under a common pause duration, for native and non-native speakers.

In order to investigate these research questions, we performed two listening experiments using excerpts from English textbooks (see General Method below for details), in which both the comma pause and the period pause were varied with the same steps (Experiment 1), or varied independently (Experiment 2). Pause durations were used in a range from 0.075 to 4.8 s. First, in order to ascertain that the selected segments were typical English speech segments, we analyzed their pause durations and the articulation rate. Following this, native-English speakers and non-native speakers (native-Chinese speakers and native-Japanese speakers) were asked to evaluate the segments on 23 items (Experiment 1) or 12 items (Experiment 2). These evaluations were then subjected to factor analysis.

## Speech Segments Used in Experiment 1 and Experiment 2

The segments used in Experiment 1 and Experiment 2 are shown in Table 1. Four English speech segments uttered by native-English speakers were extracted from English textbooks and utilized as speech materials. We chose written materials in order to be able to systematically control the stimulus conditions. The total durations of the four speech segments were 21.02, 23.02, 31.72, and 29.92 s, respectively. Speech Segments 1, 2, and 3 were used in Experiment 1 and Segments 3 and 4 were used in Experiment 2. Table 1 shows the comma- and period-pause durations for each segment. A comma pause is the pause at punctuation marks within sentences, like a comma, a semicolon, or a dash. A period pause is the pause at punctuation marks between sentences, like a period, or a question mark. The “Others” category in Table 1 are pauses mainly made for breathing. The mean pause duration for commas ranged from 0.51 to 0.78 s, while the mean pause duration for periods ranged from 1.40 to 1.43 s. The number of syllables ranged from 53 to 62, and the articulation rate of original speech segments varied from 3.04 to 3.96 syllables per second. The pause durations were comparable to the durations of commas and periods mentioned in previous studies (O’Connell and Kowal, 1986; Yamashita and Fuyuno, 2015; Liu et al., 2016; Yamashita et al., 2019). The articulation rate of the speech segments used here was a little slower than that for (American) English in daily conversation (4.88 syllables/s, Kuhnert and Antolík, 2018; 5.12 syllables/s, Jacewicz et al., 2009).

**TABLE 1:** Speech segments used in Experiment 1 and Experiment 2.

Segment 1 (Exp 1)		Author and Title: Patrick McGrath’s: “O’Malley and Schwartz”			
**Content:** “His hair hangs about his hollow, stubbled cheeks in a mess of tangled knots, and as he peers about him into the jostling throng there is in his deep-set eyes an expression of such melancholy, such sheer pain, that you would think some ghastly tragedy had befallen him, to bring him to these dire straits.”					

**Speaker**	**Number of Words**	**Number of Syllables**	**Number of Consonants**	**Average Pause Duration (s) (SD)**	**Segment Duration (s)**

male	56	72	129	Commas: 0.55 (0.23)	21.02

**Segment 2 (Exp 1)**	**Author and Title:** Gregory Bateson’s “What Science Can and Cannot Predict”				

**Content:** “According to the popular image of science, everything is, in principle, predictable and controllable; and if some event or process is not predictable and controllable in the present state of our knowledge, a little more knowledge and, especially, a little more know-how will enable us to predict and control the wild variables.”					

**Speaker**	**Number of Words**	**Number of Syllables**	**Number of Consonants**	**Average Pause Duration (s) (SD)**	**Segment Duration (s)**

male	53	91	140	Commas: 0.78 (0.33)	23.02

**Segment 3 (Exps 1, 2)**	**Author and Title:** Mary Catherine Bateson’s “Against Focused Attention”				

**Content:** “Life is complicated. It is simplifying but dangerous to have one overriding concern that makes others unimportant — rage or passion or the kind of religious exultation that seeks or inflicts martyrdom. The most striking cause of narrowed attention at the national level is warfare. In a complex world of conflicting priorities, going to war can be a tremendous relief.”					

**Speaker**	**Number of words**	**Number of syllables**	**Number of consonants**	**Average pause duration (s) (SD)**	**Segment duration (s)**

female	59	102	167	Commas: 0.60 (0.04) Periods: 1.40 (0.51) Pause Duration: 1.07 (0.44)	31.72

**Segment 4 (Exp 2)**	**Author and Title:** Gregory Bateson’s “What Science Can and Cannot Predict”				

**Content:** “Under tension, a chain will break at its weakest link. That much is predictable. What is difficult is to identify the weakest link before it breaks. The generic we can know, but the specific eludes us. Some chains are designed to break at a certain tension and at a certain link. But a good chain is homogeneous, and no prediction is possible.”					

**Speaker**	**Number of words**	**Number of syllables**	**Number of consonants**	**Average pause duration (s) (SD)**	**Segment duration (s)**

male	62	91	148	Commas: 0.51 (0.04) Periods: 1.43 (0.60) Pause Duration: 1.08 (0.65)	29.94

## Experiment 1

### Method

Experiment 1 consisted of a listening experiment in which the pause durations in three short English speech segments were varied together into the same 7 steps: 0.075, 0.15, 0.3, 0.6, 1.2, 2.4, and 4.8 s. This range included a pause duration (0.075 s) that was shorter than 0.10 s, which is considered as a minimum psychologically functional duration in reading (Hieke et al., 1983). Although Oehmen et al. (2010) utilized 0.01 as a threshold for manual segmentation in speech, it has been shown that silent intervals of 0.10 s can appear in speech not as pauses, but as silent intervals preceding stop consonants (Suen and Beddoes, 1974). In a study of silences in turn-taking from the view of conversational corpora, Heldner and Edlund (2010) used 0.18 s as the smallest pause duration to minimize the risk of confusing stop closures with pauses. Goldman-Eisler (1968) even suggested a cut-off point of 0.25 s as a threshold to separate hesitation pauses and phonetic stops. More importantly, as described above, previous research on comma- and period-pause duration show that they physically are in a range of about 300 - 1000 ms or longer (Liu et al., 2016; Yamashita et al., 2019). The longest pause duration (4.8 s) in our experiment was longer than the longest pause duration obtained with L2-learners of native Japanese speakers (Yamashita et al., 2014). The speech stimuli were rated on 23 items (see below), and factor analysis was performed over the ratings.

#### Participants

Both non-native English speakers (Chinese-native speakers, Japanese-native speakers) and native-English speakers joined the experiment. The native-English group consisted of 19 participants (5 males, 18-23 years old, average 20.8, SD = 1.9; 14 females, 18-45 years old, average 22.5, SD = 6.8). They were students or employes from the School of Psychology, National University of Ireland, Galway, Republic of Ireland. The Irish participants were English-educated from birth.

The group of non-native participants consisted of Chinese and Japanese speakers. Data were collected from 20 native-Chinese speakers (6 males, 19-33 years old, average 23.3, SD = 4.5; 14 females, 18-27 years old, average 22.2, SD = 2.3). They were undergraduate students and graduate students from 8 different universities in Beijing, People’s Republic of China (i.e., Peking University, University of International Relations, University of Science and Technology Beijing, University of Chinese Academy of Sciences, University of International Business and Economics, Beijing Jiaotong University, China University of Mining and Technology in Beijing, and Beijing Forestry University). Their majors varied from psychology, linguistics, civil engineering, cellular biology, to (applied) mathematics. They had studied English as their second language (L2) from the age of 6 to 16 years. Three had scores on the Test of English as a Foreign Language (TOEFL IBT; scores = 82-112), one had a score on the International English Language Testing System (IELTS; score = 6.5), while 17 had taken the College English Test (CET-4; scores = 452-600, CET-6; scores = 450-632). One Chinese participant had scores on two different English proficiency tests. All except one had received additional English lectures in university. The group of native-Japanese speakers consisted of 19 participants. They were students from Kyushu University, Fukuoka, Japan (13 males, 21-30 years old, average 23.8, SD = 2.52; 6 females, 21-38 years old, average 25.2, SD = 5.8). Five had taken TOEIC (scores = 450-895), one had taken IELTS (score = 7.0), three had taken TOEFL (two standard tests, scores = 350 and 450; one TOEFL ITP, score = 520). One Japanese participant had scores on two different English proficiency tests. Eleven of them had not taken any English proficiency test, but had passed the entrance exam of Kyushu University, Fukuoka, Japan, which included an English proficiency test.

All participants reported to have normal hearing. Before starting the experiment, the procedure of the experiment was explained to them. All agreed to participate and had provided written informed consent. The participants were paid for their time. The experiment was conducted with prior approval of the Ethics Committee of Kyushu University, Fukuoka, Japan; the Research Ethics Committee of the National University of Ireland, Galway; and the Human Subject Review Committee of Peking University.

#### Speech Stimuli

Three speech segments were selected as stimuli (Table 1). The first two segments (Speech Segment 1 and 2) were extracted from an English textbook for university students (Faculty of Liberal Arts, University of Tokyo English Subcommittee, 1998), which was accompanied by a compact disk with spoken texts. The segments were uttered each by a different male speaker. The third speech segment (Speech Segment 3) was extracted from another English textbook with a compact disk (Faculty of Liberal Arts, University of Tokyo English Subcommittee, 2000). It was uttered by a female speaker. The English textbooks were used in the University of Tokyo, Japan, for English education. The editors were native-English speakers from the Department of English, the University of Tokyo, Komaba, and English-education professionals.

The segments were prepared as follows. First, the speech segments were transformed from the “.cda” format and saved as “.wav” files, in order to edit the waveforms. Next, sections with sound energy (i.e., utterances) and sections without sound energy (i.e., silent sections) were semi-automatically extracted using the audio-software “Praat” (Boersma and Weenink, 2015). Using “Praat”, the speech segments were annotated to a TextGrid (Annotate function: to TextGrid (silences); guidelines for settings: Silence threshold: −35 dB; Minimum silent interval duration: 0.1 s; Minimum sounding: 0.1 s). All the utterances were then saved as separate digital samples. Following this, at temporal positions in the three original speech segments at which a comma, a period, a semicolon, or a dash appeared, a new pause duration was inserted using a program in ‘J’ programming language. Every other pause duration longer than 0.1 s was adjusted to 0 s, because we only focused on durations at punctuation marks. The pause durations that were inserted for commas and periods were fixed at 0.075, 0.15, 0.3, 0.6, 1.2, 2.4, and 4.8 s, resulting in 21 speech stimuli in total. The duration of each pause was the same for commas and for periods. The pause at the semi-colon in Speech Segment 2 spoken by the male speaker and the pause at the dash in Speech Segment 3 spoken by the female speaker were also made with the seven durations. Finally, the average intensities of the stimuli were equalized (65 dBA). Speech only from the left channel was used to make a mono speech sample, enabling easier calibration of the sound level before presentation to the participants.

#### Apparatus

The speech stimuli were diotically presented to the participants in a soundproof booth (background level < 30 dBA), by means of monitor headphones (Roland RH-300) and a USB headphone amplifier (AT-HA40USB). The stimuli were presented and controlled through an interface using a tablet (Microsoft Surface 3 64GB, OS Windows 8.1). A customized program in ‘J’-language was used to equalize the level of the stimuli. The sound pressure level was measured with a sound level meter (ACO, Type 6240), and an artificial ear (Brüel and Kjaer, 4153, Naerum, Denmark).

#### Procedure

The experiment was conducted in three different places. The data from the native-English participants were obtained in Galway, Republic of Ireland, the data from the Chinese participants were gathered in Beijing, People’s Republic of China, while the data from the Japanese participants were obtained in Fukuoka, Japan. In the soundproof booth, the stimuli were diotically presented to the participants in three sessions. In all three sessions, the participants were asked to judge the stimuli on 23 evaluation items using a 10-point rating scale from “not” (1) to “very much” (10). The evaluation items are indicated in Table 2. They were selected based on research on the relation between temporal structures of speech and listeners’ impressions of the speaker’s personality (Uchida, 2005). items, originally in Japanese and translated into English and Chinese for the speakers of those languages, consisted of 16 positive adjectives, like “fluent,” “natural,” and “skillful,” 4 negative adjectives (“shrill,” “nervous,” “rushed,” and “rough-timbred”), and 3 neutral/negative items (“speedy,” “high-pitched,” and “fast”).

**TABLE 2:** Evaluation items used in Experiment 1 and Experiment 2, as judged by native-English speakers and non-native speakers (native-Chinese and native-Japanese).

Experiment 1	Experiment 1, Experiment 2
“intelligible,” “polite,” “dynamic,” “clear-cut,” “elegant,” “smooth,” “nervous,” “experienced,” “shrill,” “fluent,” “easy to understand”	“with appropriate rhythm,” “rushed,” “natural” “rough-timbred,” “skillful,” “speedy,” “at a suitable tempo,” “well-practiced,” “fast,” “with appropriate pause duration,” “friendly,” “high-pitched”

The stimuli were presented to the participants through headphones, 0.5 s after the participant pressed the “PLAY” button on the interface. When stimulus presentation was finished, the participants rated the stimulus on the 23 evaluation items, using pen and paper on which the 10-point rating scales were indicated. There was no time limit for participants to give each rating; the experiment was self-paced. Before the experiment, there were 7 practice trials, randomly chosen from the three speech segments. The results of these practice trials were not considered for further analysis. The participants could take a break following practice. The experiment was divided into two sessions, with the second session following the first, with a break in between. There were 12 trials in the first session, and 11 trials in the second session. The first trial and the last trial in each session were the same, but the results of the first trial were not analyzed. In total, rating data were obtained from 21 speech stimuli (3 speech segments × 7 durations). The experiment took approximately 50 min. After the last session, the participants were asked to complete a questionnaire about their personal details and language background.

### Results

The results were analyzed in the following steps. In order to check whether the rating data were suitable for factor analysis, Kaiser-Meyer-Olkin (KMO) tests were performed. The results showed that the sampling was adequate overall for the data obtained from the native-English listeners (0.947), the Chinese listeners (0.944), and the Japanese listeners (0.934). [Bartlett’s tests of sphericity were all significant (*p* < 0.001)]. Following principal component analysis (PCA) with varimax rotation, four factors were extracted for all three language groups. The factors were labeled according to the categorical items, following Pett et al. (2003). The first factor was called the “Speech Naturalness factor.” In this factor, the evaluation items “elegant,” “skillful,” “smooth,” “with appropriate rhythm,” “natural,” “experienced,” “well-practiced,” “with appropriate pause duration,” “at a suitable tempo,” “polite,” “friendly,” “fluent,” “intelligible,” and “easy to understand” were included for all three language groups. The second factor could be summarized as the “Speech Rate factor”; it included evaluation items “speedy,” “rushed,” and “fast” for all three language groups. The third factor (“high-pitched,” “shrill”) and the fourth factor (“rough-timbred”) related to sound quality. The cumulative percentages of variance at the third and fourth factor were in between 66 and 74%, in all of the three language groups. The first (Speech Naturalness) and the second factor (Speech Rate) were taken into further consideration, because their cumulative percentage of variance was about 60% for all three language groups.

**FIGURE 1:**
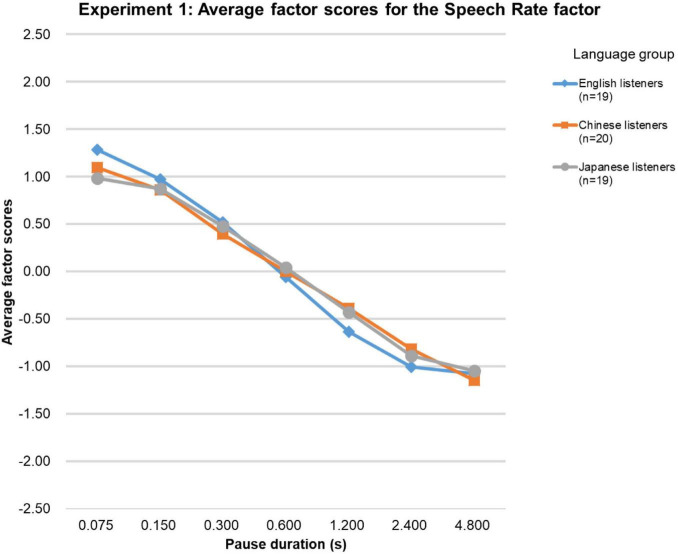
Results of Experiment 1. The average factor scores for the Speech Rate factor.

Figure 1 shows the average factor scores for the Speech Rate factor. Since Shapiro-Wilk tests showed that the factor scores were not normally distributed for all three language groups, comparisons of factor scores were performed with Friedman tests (*p* < 0.05), followed by pair-wise Wilcoxon tests with Holm-Bonferroni correction for multiple comparisons. For all three language groups, the Friedman tests were significant [native-English group (χ2 (df = 6, *n* = 19) = 104.4, *p* < 0.0001; Chinese group (χ2 (df = 6, *n* = 20) = 111.3, *p* < 0.0001; Japanese group (χ2 (df = 6, *n* = 19) = 97.3, *p* < 0.0001]. Overall, paired comparisons showed that the factor scores significantly decreased as pause duration increased. There were only two exceptions. The difference between the factor scores for the stimuli with the 2.4-s and the 4.8-s pause durations was not significant in the native-English group, while in the Japanese language group, the difference between the stimuli with the 0.075-s and the 0.15-s pause durations was not significant. The Kendall’s Coefficient of Concordance test showed that the factor scores obtained for the three language groups were highly similar (Kendall’s W = 1.00, *p* < 0.01, *n* = 3, *k* = 7).

**FIGURE 2:**
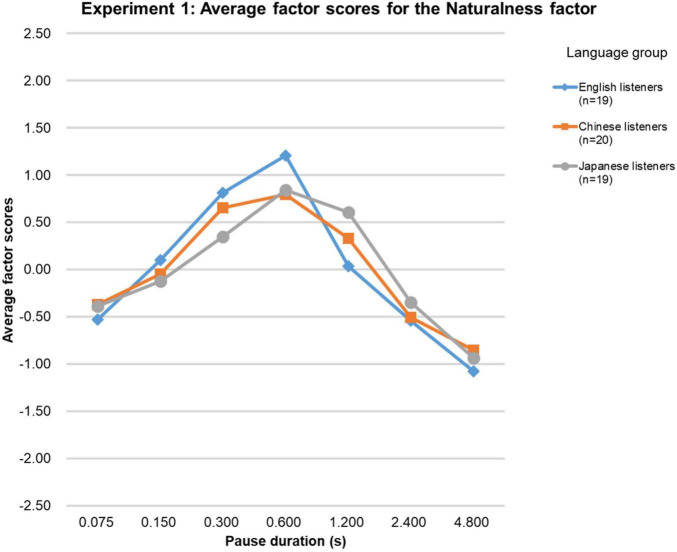
Results of Experiment 1. The average factor scores for the Naturalness factor.

Figure 2 shows the average factor scores for the Naturalness factor. The Kendall’s Coefficient of Concordance test showed that the factor scores for the three language groups were very similar for this factor as well (Kendall’s W = 0.94, *p* < 0.01, *n* = 3, *k* = 7). Since the factor scores for the native-English group were not normally distributed, again Friedman tests with Holm-Bonferroni correction were performed over factor scores. For the Naturalness factor the test results were significant for all three language groups [native-English group (χ2 (df = 6, *n* = 19) = 93.3, *p* < 0.0001; Chinese group (χ2 (df = 6, *n* = 20) = 92.5, *p* < 0.0001; Japanese group (χ2 (df = 6, *n* = 19) = 73.9, *p* < 0.0001]. For the native-English group, the factor score was significantly higher than that for any of the other stimuli. For the Chinese group, only the factor score for the 0.3-s stimuli was not significantly higher than that for the 0.6-s stimuli. For the Japanese group, the factor score for the 0.6-s stimuli was not significantly higher than that for the 1.2-s stimuli. In conclusion, the Naturalness factor scores for the stimuli with the 0.6-s pause duration were the highest in all three groups.

### Discussion

Factor analysis over the rating data revealed two noteworthy results. First, for all three language groups, the factor scores for the Speech Rate factor (Figure 1) decreased as pause duration increased. Although the physical speech rate (i.e., the articulation rate) of the utterances used in Experiment 1 was the same, the listeners perceived a decrease in the overall speech rate with an increase only in pause duration. In the preliminary research with Japanese L2-learners of English described in the introduction (Yamashita et al., 2014; Liu et al., 2016), a significant negative correlation between speech rate and pause duration was found. The results of Experiment 1 reflected this in the large-scale experiment with three language groups, including native-English participants.

As for the Naturalness factor, the pause duration of 0.6 s received the highest factor scores. However, there were slight differences between language groups. For example, the difference between the 0.6-s stimuli and the other pause duration conditions was more pronounced for the native-English group than for the non-native groups. Factor scores for stimuli with relatively short (0.075 and 0.15 s) and long (2.4 and 4.8 s) pause durations received the lowest scores.

The results of Experiment 1 thus suggest that just changing the pause duration for commas and periods in the same duration can change the subjective impression systematically, affecting the perceived speech rate and speech naturalness. A pause duration of 0.6 s seemed to make speech natural for both native-English and non-native listeners. This duration is also a good index for music tempo (Fraisse, 1982), suggesting a commonality in the perception of temporal properties of music and speech. One limitation of the present experiment, however, was that the pause duration was fixed for each punctuation mark, while previous research has shown that the physical duration of periods in spoken texts is approximately twice as long as that of commas, as mentioned in the introduction (O’Connell and Kowal, 1986; Yamashita and Fuyuno, 2015; Liu et al., 2016; Yamashita et al., 2019). Therefore, in order to further investigate how pause duration influences the subjective impression of speech, in Experiment 2 the comma- and the period-pause duration were varied independently.

## Experiment 2

### Method

In Experiment 2, comma- and period-pause durations were manipulated separately, in 7 steps varying from 0.15 to 2.4 s. In this experiment, the original speech and speech without pauses were also included, as control conditions. Similar to Experiment 1, the participants were native-English and non-native English speakers (native-Japanese and native-Chinese speakers). We investigated how the listeners’ impressions would change as a function of pause duration by collecting rating scale data for 12 evaluation items (see below), which were then subjected to factor analysis.

#### Participants

Three participant groups consisted of native-English speakers and Chinese and Japanese non-native English speakers. The native-English group consisted of 24 participants (18 students from the National University of Ireland, Galway, Republic of Ireland, and 6 students or English-education professionals in Fukuoka, Japan). They were 14 males (18-49 years old, average 27.0, SD = 9.6) and 10 females (19-39 years old, average 22.2, SD = 5.7). The Irish participants were English-educated from birth, and one of them had participated in Experiment 1. The Chinese non-native group consisted of 20 native-Mandarin Chinese speakers. They were students from Kyushu University, Fukuoka, Japan (8 males, 23-34 years old, average 26.5, SD = 3.1; 12 females, 19-26 years old, average 23.8, SD = 1.7). The Japanese non-native group consisted of 20 native-Japanese speakers. They were also students from Kyushu University, Fukuoka, Japan (10 males, 21-25 years old, average 22.6, SD = 1.4; 10 females, 20-22 years old, average 21.6, SD = 0.7).

Out of the 20 Chinese participants, 4 had scores on the Test of English as a Foreign Language (TOEFL IBT; scores = 85-99), 8 had scores on the Test of English for International Communication (TOEIC; scores = 630-885), 4 had scores on the International English Language Testing System (IELTS; scores = 6.0-7.5), while 14 had taken the College English Test (CET-4; scores = 440-500, CET-6; scores = 450-600). Eight Chinese participants had scores on two different English proficiency tests, while one had three different English certificates. None of them had participated in Experiment 1. From the 20 Japanese participants, 10 had taken TOEIC (scores = 480-895), one had taken IELTS (score = 7.0), one had taken TOEFL ITP (score = 500), and 4 had completed TOEFL (scores = 400-600). Two students had taken two tests, while 6 had not taken any English proficiency test yet, but had passed the entrance exam of Kyushu University, Fukuoka, Japan, which includes an English proficiency test. Two of them had participated in Experiment 1. All participants reported to have normal hearing and were paid for their time. All agreed to participate and provided written informed consent, after the procedure of the experiment was explained to them. The experiment was conducted with prior approval of the Ethics Committee of Kyushu University, Fukuoka, Japan and the Research Ethics Committee of the National University of Ireland, Galway.

#### Speech Stimuli and Apparatus

Two English speech segments (Speech Segment 3 and Speech Segment 4, Table 1) were selected as stimuli. One speech segment was the same as in Experiment 1 (Speech Segment 3), spoken by a female speaker. The other segment (Speech Segment 4) was newly extracted from Faculty of Liberal Arts, University of Tokyo English Subcommittee (1998), which was uttered by a male speaker. The stimulus preparation was the same as in Experiment 1. The pause durations were 0.15, 0.3, 0.6, 1.2, and 2.4 s, and the comma duration and the period duration were varied independently, resulting in 25 stimuli for each segment. Furthermore, different from Experiment 1, for both speech segments stimuli without any pauses were made for a control condition and the original speech segments with the pause durations as uttered by the male or the female speaker were used as well. The original speech segments included other pauses where there was no punctuation mark. In total, 54 stimuli were used in the experiment, and the average presentation levels of the stimuli were equalized (65 dBA). The same apparatus was used as in Experiment 1.

#### Procedure

The experiment was conducted in two different places. The data from the native-English participants were obtained in Galway, Republic of Ireland, and in Fukuoka, Japan. The data from the Chinese and Japanese participants were obtained in Fukuoka, Japan. The procedure was the same as in Experiment 1, except that, in this experiment the participants rated the stimuli in three sessions on 12 evaluation items (Table 2). These 12 items were also used in Experiment 1. Because the comma- and period-pause durations were varied independently in the present experiment, fewer items were used to limit the total task duration. The first session was a short practice session. In the practice session, Speech Segments 3 and 4 (Table 1) were presented, each with a comma- and period-pause duration of 0.6 s. These stimuli were the same for all participants, and the data were not used for further analysis. After the practice session was completed, two experimental sessions were carried out. In each session, 28 stimuli were randomly presented. The first stimulus and the last stimulus were the same, but the results of the first were not analyzed.

The stimuli were diotically presented to the participants through headphones 0.5 s after the participant pressed the “PLAY” button on the interface. When stimulus presentation was finished, the participants evaluated the stimulus on the 12 evaluation items, using pen and paper on which the 10-point rating scales were indicated. There was no time limit for participants to give each rating; the experiment was self-paced and took 75 min, approximately. One limitation of Experiment 1 was also that the English proficiency of the non-native participants was checked only by asking whether they had actually performed an English proficiency test. In order to ascertain the English proficiency of the non-native participants, additional English listening and grammar tests were conducted after the last session. That is, the participants were asked to write down the contents of the two speech stimuli used in the experiment, i.e., the spoken content of the female speaker (Speech Segment 3) and the male speaker (Speech Segment 4), as well as 5 randomly-selected sentences, each uttered by a different speaker, from an English-speech database consisting of short sentences (NTT-AT, 2002). To test English grammar knowledge, previous English-proficiency questions of the entrance exam of Kyushu University, Fukuoka, Japan, were used as well. All the participants (both Chinese and Japanese participants’ groups) could answer at least 70% of all the English questions. From this we assumed they had sufficient English capacity to participate in this listening experiment.

### Results

The results were analyzed using the same protocol as used in Experiment 1. Since three of the native-English participants did not evaluate the stimuli on three or more evaluation items, their data were not analyzed. Five native-English participants had missed one evaluation item, and 7 had provided no score on two evaluation items. Their data were nevertheless included in the PCA; instead of the blank data entry we added the median score of the rating scale (5.5). Before performing PCA, KMO-tests showed that the data sampling was adequate overall for the native-English participants (0.852), the Chinese participants (0.877), and the Japanese participants (0.914). [Bartlett’s tests of sphericity were also all significant (*p* < 0.001)].

For the native-English and the Chinese language group, three factors were extracted from PCA with varimax rotation, and two factors for the Japanese language group. Similar to the results of Experiment 1, for all three language groups, the first factor could be interpreted as the Speech Naturalness factor and the second factor as the Speech Rate factor. The Speech Naturalness factor included the evaluation items “with appropriate rhythm,” “at a suitable tempo,” “natural,” “with appropriate pause duration,” “skillful,” “well-practiced,” and “friendly.” The Speech Rate factor included “speedy,” “rushed,” and “fast” for all three language groups. Also similar to Experiment 1, the third factor that appeared in the PCA for the native-English and the Chinese language group related to sound quality. The cumulative percentages of variance for the Speech Naturalness and the Speech Rate factor were over 60% for all three language groups. The cumulative percentage of variance at the third factor in the English and Chinese language group reached 71%. Only the Speech Naturalness and the Speech Rate factor were discussed here.

**FIGURE 3:**
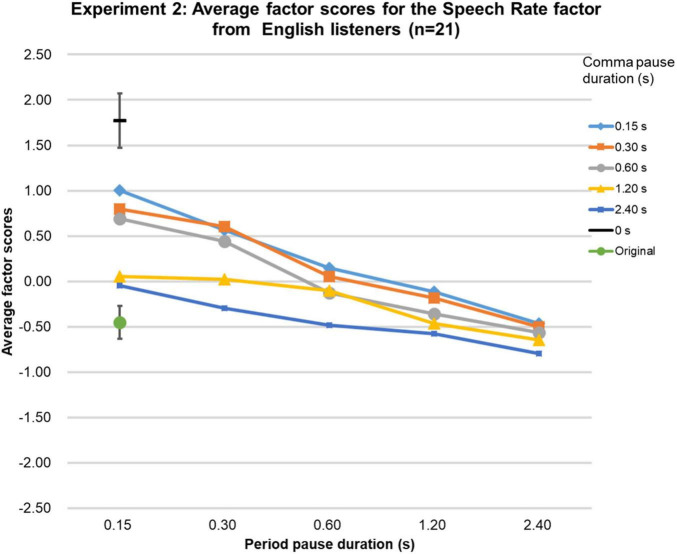
Results of Experiment 2. The average factor scores for the Speech Rate factor from native-English participants (*n* = 21). The error bar shows the 95%-confidence intervals.

**FIGURE 4:**
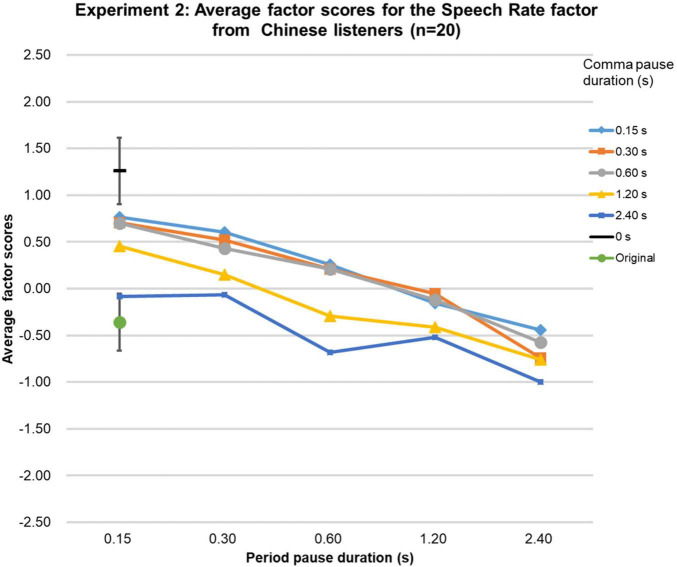
Results of Experiment 2. The average factor scores for the Speech Rate factor from Chinese participants (*n* = 20). The error bar shows the 95%-confidence intervals.

**FIGURE 5:**
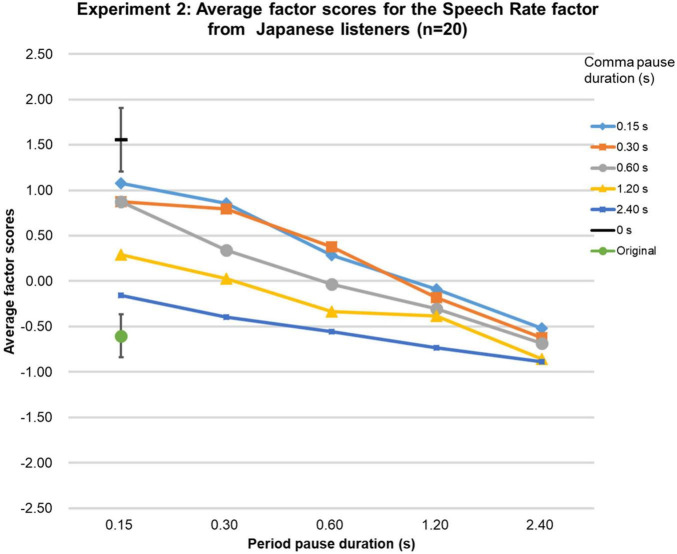
Results of Experiment 2. The average factor scores for the Speech Rate factor from Japanese participants (*n* = 20). The error bar shows the 95%-confidence intervals.

Because Shapiro-Wilk tests showed that the Speech Rate factor scores were not normally distributed for all three language groups, they were analyzed as in Experiment 1. The Friedman tests were significant [native-English group (χ2 (df = 26, *n* = 21) = 281.6, *p* < 0.0001; Chinese group (χ2 (df = 26, *n* = 20) = 323.5, *p* < 0.0001; Japanese group (χ2 (df = 26, *n* = 20) = 395.5, *p* < 0.0001], and the Kendall’s Coefficient of Concordance test showed that the factor scores obtained were highly similar among the groups (Kendall’s W = 0.98, *p* < 0.01, *n* = 3, *k* = 27). The factor scores for the original speech were as expected based on the physical durations of the speech stimuli as shown in Figures 3–5. Based on the 95%-confidence intervals in Figures 3–5, for all three language groups, stimuli with a period-pause duration below 1.2 s and a comma-pause duration below 0.6 s had significantly higher factor scores than the original speech. Similar to Experiment 1, the results clearly show that when the comma- and period-pause duration became longer, the listeners in all three language groups perceived a slower speech rate, even though only the pause duration was adjusted and not the speech itself. The average Speech Rate factor scores showed a steady decrease as pause duration increased from 0.15 to 2.4 s for all three groups. The average factor score for the 0-s condition was, as expected, the highest. Overall, Experiment 2 confirmed that the perceived speech rate thus can be influenced by only manipulating pause duration.

**FIGURE 6:**
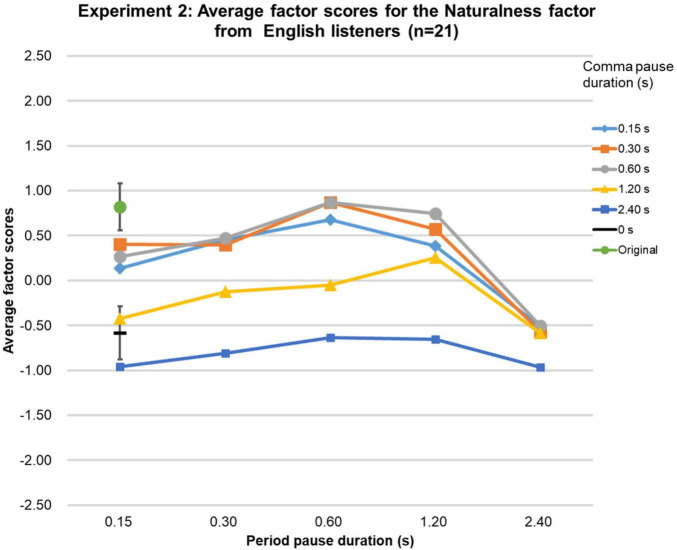
Results of Experiment 2. The average factor scores for the Naturalness factor from native-English participants (*n* = 21). The error bar shows the 95%-confidence intervals.

**FIGURE 7:**
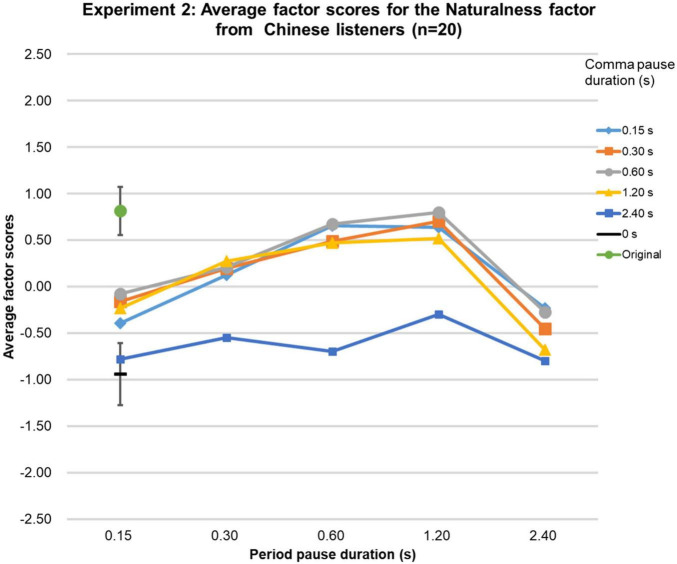
Results of Experiment 2. The average factor scores for the Naturalness factor from Chinese participants (*n* = 20). The error bar shows the 95%-confidence intervals.

**FIGURE 8:**
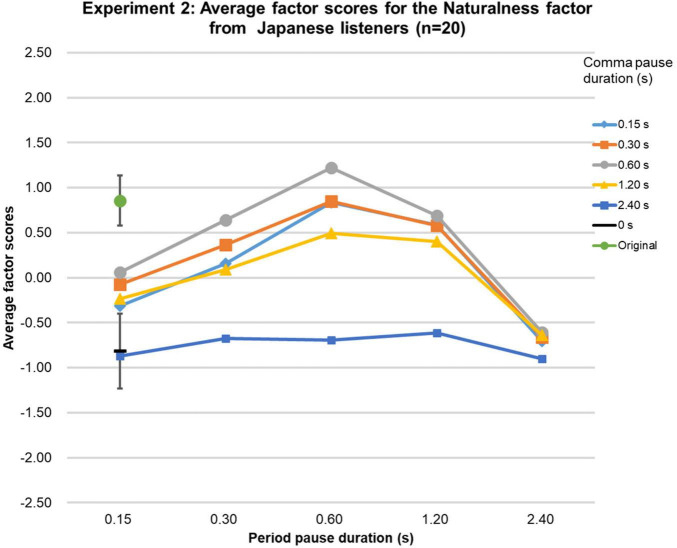
Results of Experiment 2. The average factor scores for the Naturalness factor from Japanese participants (*n* = 20). The error bar shows the 95%-confidence intervals.

The Friedman tests over the factor scores of the Naturalness factor were significant [native-English group (χ2 (df = 26, *n* = 21) = 328.7, *p* < 0.0001; Chinese group (χ2 (df = 26, *n* = 20) = 289.3, *p* < 0.0001; Japanese group (χ2 (df = 26, *n* = 20) = 336.2, *p* < 0.0001], and highly similar for the three language groups (Kendall’s W = 0.95, *p* < 0.01, *n* = 3, *k* = 27), as can be seen in Figures 6–8. In the native-English group, the factor score for speech sentences with a comma-pause duration and a period-pause duration of 0.6 s was the highest (0.87), slightly above the factor score for sentences with a comma-pause of 0.3 s and a period-pause of 0.6 s (0.84). Remarkably, these factor scores were higher than that for the original speech (0.82). For the Chinese language group, the original speech got the highest factor score (0.81), closely followed by the speech sentences with a comma-pause duration of 0.6 s and a period-pause duration of 1.2 s (0.79). For the Japanese language group, the factor score for speech sentences with a comma- and period-pause duration of 0.6 s was the highest (1.20), again, surprisingly, exceeding the factor score obtained for the original speech (0.85). From the 95%-confidence intervals in Figures 6–8, it can be seen that there were no significant differences between the factor scores for the original speech and the stimuli with adequately manipulated pause durations, mentioned above.

### Discussion

In this experiment, comma- and period-pause durations were varied independently. In the same way as in Experiment 1, factor analysis yielded two main factors, the Speech Rate factor and the Naturalness factor. The average factor scores for the Speech Rate factor showed that even if the speed of the utterances was not physically changed, the perceived speed of the sentences was affected by just changing the pause duration. If the pause duration lengthened, the listeners would hear slowed-down speech. The factor scores of the original speech clearly reflected the physical comma- and period-pause durations as measured in the original speech segments, showing the validity of the present analysis consisting of factor analysis over rating scale data.

Figures 6–8, showing factor scores for the Naturalness factor, indicate that a number of conditions of pause durations yielded speech that was as natural as the original speech. Depending on the language group, the highest scores were obtained when the comma- and period-pause duration were at a ratio of 1:2 (0.3:0.6 s; 0.6:1.2 s) or at a ratio of 1:1 (0.6:0.6 s). Thus, remarkably, even though the 1:2 ratio predominantly occurs in natural speech (O’Connell and Kowal, 1986; Yamashita and Fuyuno, 2015; Liu et al., 2016; Yamashita et al., 2019), as also is the case in our original speech segments (Table 1), the same degree of speech naturalness could be obtained with the same comma- and period-pause duration of 0.6 s. Factor scores for this condition did not significantly differ from those for the original speech. In fact, for the English and the Japanese group, the factor scores for the 0.6-s pause duration conditions were even higher than those for the original speech, which seems to suggest common cross-cultural preference for this pause duration in English speech.

## General Discussion

Two listening experiments were carried out to investigate influences of pause duration on speech impressions of English speech segments, which originally had physical pause durations typical for spoken English (Table 1). In Experiment 1, all pauses were removed, and after that all punctuated pauses (comma- and period-pauses) were remade with the same duration, between 0.075-4.8 s. In Experiment 2, comma- and period-pause durations were varied independently, in between 0.15-2.4 s. Speech segments without any pauses, as well as the original speech segments, were included as control conditions. Both native- and non-native (Chinese and Japanese) English speakers rated the segments on a broad range of items, which were then subjected to factor analysis.

In both experiments, the same two factors were observed in all language groups. These factors were interpreted as representing speech naturalness and speech rate. It is to be noted that the same two factors appeared in our preliminary study with Mandarin Chinese as evaluated by native-Chinese speakers (Lin et al., 2021). With regard to our research questions, i.e., how systematic changes in pause duration would influence subjective impressions of English speech, and whether favorable impressions occur under a common pause duration, for native and non-native speakers, we observed the following. First, the perceived speech rate decreased when the physical pause duration increased. This is in line with the results of reading task experiments, in which the speech rate and the frequency and duration of pauses are interdependent (Grosjean et al., 1979). The results are also in line with those from a study on pause function in production and perception in Japanese discourse (Sugito, 1990). Also in discourse, speech without pause sounded fast-paced, and changing the pause duration influenced the listeners’ perception of speech rate.

Second, although the physical comma- and period-pause duration in natural speech is typically 1:2 (O’Connell and Kowal, 1986; Yamashita and Fuyuno, 2015; Liu et al., 2016; Yamashita et al., 2019), the factor scores for the Naturalness factor showed that even when the comma- and the period-pause duration were equal (= 0.6 s), naturalness was very similar to – or even higher than – that of the original speech for all three language groups. In studies of time perception, durations around 0.6-0.7 s are considered as neither long nor short (Fraisse, 1964). Perceptually, the pause duration of 0.6 s therefore might be considered as natural also in English speech, regardless of the listener’s language background.

We anticipate that the perceived naturalness at an equal comma- and period-duration of 0.6 s is of use in training L2-speakers of English, for example those whose native tongue is not a stress language, because they can simply be instructed to use the same pause duration when delivering speeches in English; pausing is easier to acquire and to control than pronunciation (Matzinger et al., 2020). Furthermore, the present results may assist developments in artificial speech technology, regarding both speech generation and recognition. Further research, is necessary in order to clarify whether the 0.6-s pause duration is natural for other languages as well. Our study with Mandarin Chinese showed that speech segments with a comma-pause duration of 0.6 s, along with a period-pause duration of 0.6 s or 1.2 s received the highest scores for the Naturalness factor, and these were not significantly different from the factor scores for the original speech (Lin et al., 2021). Our study is also limited in that pause durations in other tonal languages or a mora-based language (Japanese) need to be investigated as well. Finally, it is still unclear whether the natural pause duration depends on the difficulty level of the English content, or on whether the speaker is a native English speaker or not.

## Data Availability Statement

The raw data supporting the conclusions of this article will be made available by the authors, without undue reservation.

## Ethics Statement

The studies involving human participants were reviewed and approved by the Ethics Committee of Kyushu University, Fukuoka, Japan; the Research Ethics Committee of the National University of Ireland, Galway, Ireland; and the Human Subject Review Committee of Peking University, China. The patients/participants provided their written informed consent to participate in this study.

## Author Contributions

SL, YN, and GR contributed to the conception of the study, which was supported as an international project by ME, SA, and LC. YN, LC, ME, and GR facilitated the experiments. SL, SA, and MK recruted the participants. SL and MK ran the experiments. SL, YN, and GR performed statistical analyses, and wrote the first draft of the manuscript, which was checked and improved by all authors before the first submission.

## Conflict of Interest

Author YN is employed by Sound Corporation, Fukuoka, Japan. The remaining authors declare that the research was conducted in the absence of any commercial or financial relationships that could be construed as a potential conflict of interest.

## Publisher’s Note

All claims expressed in this article are solely those of the authors and do not necessarily represent those of their affiliated organizations, or those of the publisher, the editors and the reviewers. Any product that may be evaluated in this article, or claim that may be made by its manufacturer, is not guaranteed or endorsed by the publisher.
